# Fibrin Glue Enhances Adipose-Derived Stromal Cell Cytokine Secretion and Survival Conferring Accelerated Diabetic Wound Healing

**DOI:** 10.1155/2018/1353085

**Published:** 2018-12-19

**Authors:** Ursula Hopfner, Matthias M. Aitzetmueller, Philipp Neßbach, Michael S. Hu, Hans-Guenther Machens, Zeshaan N. Maan, Dominik Duscher

**Affiliations:** ^1^Department for Plastic Surgery and Hand Surgery, Klinikum rechts der Isar, Technical University of Munich, Germany; ^2^Department for Plastic Surgery, University of Pittsburgh, Pennsylvania, USA; ^3^Hagey Laboratory for Pediatric Regenerative Medicine, Stanford University School of Medicine, Stanford, CA, USA; ^4^Division for Plastic and Reconstructive Surgery, Kepler University Hospital, Linz, Austria

## Abstract

**Introduction:**

Although chronic wounds are a major personal and economic burden, treatment options are still limited. Among those options, adipose-derived stromal cell- (ASC-) based therapies rank as a promising approach but are restricted by the harsh wound environment. Here we use a commercially available fibrin glue to provide a deliverable niche for ASCs in chronic wounds.

**Material and Methods:**

To investigate the *in vitro* effect of fibrin glue, cultivation experiments were performed and key cytokines for regeneration were quantified. By using an established murine chronic diabetic wound-healing model, we evaluated the influence of fibrin glue spray seeding on cell survival (In Vivo Imaging System, IVIS), wound healing (wound closure kinetics), and neovascularization of healed wounds (CD31 immunohistochemistry).

**Results:**

Fibrin glue seeding leads to a significantly enhanced secretion of key cytokines (SDF-1, bFGF, and MMP-2) of human ASCs *in vitro*. IVIS imaging showed a significantly prolonged murine ASC survival in diabetic wounds and significantly accelerated complete wound closure in the fibrin glue seeded group. CD31 immunohistochemistry revealed significantly more neovascularization in healed wounds treated with ASCs spray seeded in fibrin glue vs. ASC injected into the wound bed.

**Conclusion:**

Although several vehicles have shown to successfully act as cell carrier systems in preclinical trials, regulatory issues have prohibited clinical usage for chronic wounds. By demonstrating the ability of fibrin glue to act as a carrier vehicle for ASCs, while simultaneously enhancing cellular regenerative function and viability, this study is a proponent of clinical translation for ASC-based therapies.

## 1. Introduction

Chronic wounds represent a major healthcare issue, significantly affecting the quality of life of the individual and placing a substantial economic burden on the society, annually affecting 6.5 million people and costing more than 25 billion dollars in USA alone [1]. This has been accentuated by an increasingly aging population and has directed research towards both efficient prevention and effective treatments of chronic wounds [1]. Although 1126 clinical trials have been carried out to address wound healing [2], a definitive breakthrough remains elusive.

Within the last few years, stem cell therapies have emerged as a promising approach to address nonhealing wounds [3]. Bone marrow-derived stem cells (BMSCs), adipose-derived stem cells (ASCs), and induced pluripotent stem cells (iPSCs) have been successfully used to improve wound healing [4–6]. Among these, ASCs represent the most versatile cell source for tissue regeneration, due to the ease of harvesting by liposuction and relatively low ethical and processing limitations. ASCs accelerate wound closure by secreting proangiogenic and chemotactic factors, thereby influencing cells in both the wound environment and from the circulation [3, 7–10]. Additionally, due to their multipotency, ASCs have the ability to differentiate into a broad range of cell linages and potentially enhance wound closure through this mechanism as well [9, 11]. Nevertheless, the use of ASCs is still limited by several factors. Aging and diabetes have been shown to significantly diminish ASC function, particularly their wound-healing ability [3, 12, 13]. Due to the fact that chronic wounds usually occur in older patients and are associated with comorbidities such as diabetes, the efficacy of stem cell therapies in the patient cohort that needs them the most is dramatically reduced. Therefore, it is essential to optimize the delivery of ASCs and thereby enhance cell survival and key cytokine secretion.

Chronic wounds represent a harsh and cytotoxic environment, mainly characterized by inflammation and poor blood supply [14]. Ameliorating the environment with a therapeutic vehicle represents one way of prolonging transplanted cell viability. Carrier and delivery vehicles such as hydrogels are a possibility to initially provide a cell niche and promote ASC survival. Although many hydrogels like chitosan, agarose, alginate, gelatin, or collagen have been used in preclinical as well as clinical studies, none are routinely used in clinical practice [15–19]. Fibrin, commonly used in clinical practice for its hemostatic and adhesive properties [20, 21] has recently been investigated as carriers for cells. Mogford et al. successfully used a mixture of fibrin, platelet-derived growth factor, and fibroblasts to enhance wound closure [22]. Zimmerlin et al. showed *in vitro* that the stromal vascular fraction (SVF) composed of endothelial cells, fibroblasts, ASCs, and immune cells can successfully be delivered using a fibrin glue [23]. In order to better understand the effects of fibrin glue on ASC function, we used the commercially available Tisseel fibrin glue as a delivery vehicle for ASCs and evaluated its effect on ASC cytokine profile and efficacy for diabetic wound healing.

## 2. Material and Methods

### 2.1. Expansion of Cells

ASCs from three different healthy donors were purchased from Lonza Group (Basel, Switzerland). ASCs were expanded under standard cell culture conditions in a StemMACS expansion medium (Miltenyi Biotec, Bergisch Gladbach, Germany).

### 2.2. Experimental Set-up

For experimental seeding, ASCs were pooled and counted with Casy TT Counter (Omni Life science, Bremen, Germany). Three different conditions were performed in triplicates. For standard cell culture condition 1 × 10^6^ ASCs were resuspended in 8 ml StemMACS expansion medium and seeded in 10 cm dishes.

Cell culture in fibrin glue was performed in Tisseel (Baxter, Illinois, USA). Both components, fibrin and thrombin, were prediluted 1 : 4 in a StemMACS expansion medium. 1 × 10^6^ ASCs were resuspended in 4 ml prediluted thrombin, transferred in a 10 cm dish, and then mixed with 4 ml prediluted fibrin.

As an additional control, fibrin glue without cells (prepared identically to as described above) was included in the experimental set-up. After cell seeding, all dishes were filled with 24 ml StemMACS expansion medium and cultivated 1 week under standard cell culture conditions. After one week, supernatant was aspirated and centrifuged to obtain a cell-free material for analysis. Cell-free supernatant was aliquoted and stored at −80°C.

### 2.3. Protein Analysis

To examine protein expression of stromal cell-derived factor 1 (SDF-1), basic fibroblast growth factor (bFGF/FGF2) and matrix metalloproteinase-2 (MMP-2) ELISAs (all DuoSet, R&D Systems, Minnesota, USA) were performed according to the manufacturer's instructions.

### 2.4. Wound-Healing Model

All animal experiments were carried out in accordance with the institutional animal guidelines.

All mice were ordered from Charles River Laboratories, Wilmington, MA, USA, (http://www.criver.com). Db/db mice and luciferase positive mice were randomized to three different groups. Group one received treatment with fibrin glue seeded with ASCs, group two received ASCs injected with PBS, and group three received fibrin glue alone.

At the dorsum of each mouse, two full-thickness excisional wounds with a diameter of 6 mm were created and splinted by silicone rings. The silicone rings were stably sutured with 6-0 nylon sutures for the prevention of wound contraction.

After treatment, wound beds were covered with an occlusive dressing (Tegaderm; 3M, St. Paul, MN, http://www.3m.com). Documentation was performed on days 0, 4, 8, 12, 16, 18, and 20 by digital photography. Wound area was measured by using ImageJ software (NIH).

### 2.5. *In Vivo* Imaging System (IVIS)

Bioluminescent imaging (BLI) was used to assess viability and location of transplanted ASCs *in vivo.* The IVIS was performed by initial mouse anesthesia by 2.5% isoflurane, followed by a 200 *μ*l intraperitoneal injection of D-luciferin. Using an IVIS Spectrum System (Caliper Life Sciences, Hopkinton, MA), the mice were continuously anaesthetized by 1.5% isoflurane, and images were acquired at a 60-second exposure until peak signal was reached. Radiance was quantified in photons per second per centimeter squared per steradian [24]. ASCs survival was evaluated on days 0, 3, 5, 7, and 9.

### 2.6. Assessment of Wound Vascularity

Vascularity of healed wounds (*n* = 8 wounds) was assessed by immunohistochemical staining for CD31 (endothelial cell marker). Wounds were harvested immediately upon closure and processed for paraffin sectioning at a thickness of 7 *μ*m. After deparaffinization, slides were washed with PBS and then blocked in a humidified chamber for 2 hours. Primary antibody at 1 : 100 dilution was used (Rb *α* CD31, Ab28364, Abcam, Cambridge, United Kingdom, http://www.abcam.com). Slides were incubated with the primary antibody overnight at 4°C. Secondary antibody was applied and incubated for 4 hours at room temperature (AF547 Goat *α* Rabbit, Life Technologies, Grand Island, New York, http://www.lifetechnologies.com). Additionally, nuclei were stained with DAPI (4′6′-Diamin-2-Phenylindol).

For quantification ImageJ was used with automatic threshold. By binarizing the fluorescence signal of each pixel, we determined the “pixel-positive area” and then evaluated the total fluorescence signal of each slide.

### 2.7. Statistical Analysis

For comparison between groups, we used Student's *T*-Test. Due to the fact, we compared multiple groups, and we corrected the result with the Bonferroni method. All data are shown as mean ± standard error of the mean (SEM). A *p* value of <0.05 was considered to be statistically significant.

## 3. Results

### 3.1. Fibrin Modulates ASC Cytokine Secretion

**Figure 1:**
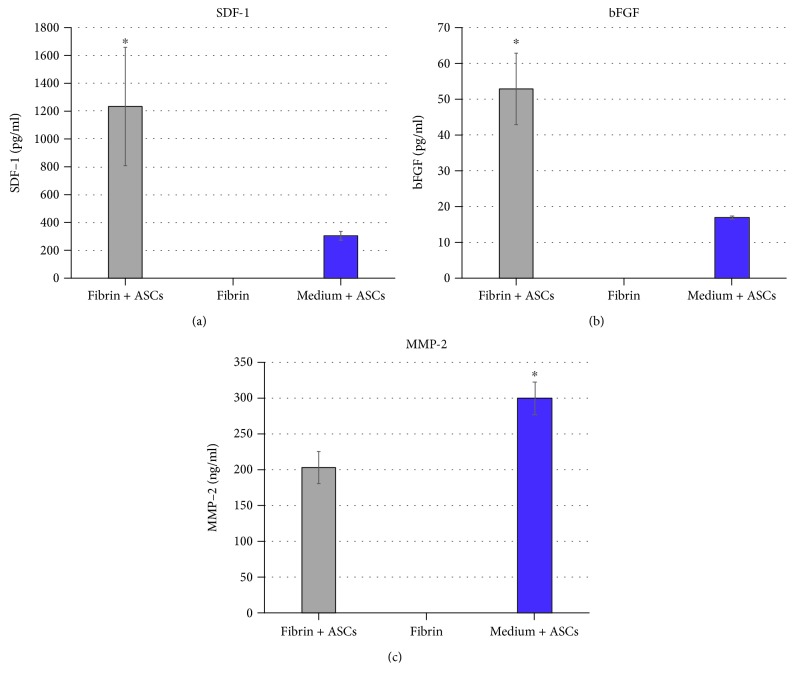
Key protein secretion of fibrin- and medium-delivered ASCs. Expression of key proteins was significantly enhanced in the fibrin group (means, ±SEM), when comparing to ASCs expanded in a medium (mean, ±SEM) and negative control (mean, ±SEM).

The quantity of proregenerative cytokines in ASCs seeded with fibrin glue was significantly higher than the group with ASCs seeded in media alone or fibrin alone (Figure 1). The secretion of bFGF (*p* = 0.0034) and SDF-1 (*p* = 0.0195) was significantly increased in the fibrin/ASC group. MMP-2, the matrix metalloproteinase-2, showed significantly lower levels in the fibrin/ASC group (*p* = 0.0066).

### 3.2. Fibrin Glue Significantly Prolongs *In Vivo* ASC Survival

**Figure 2:**
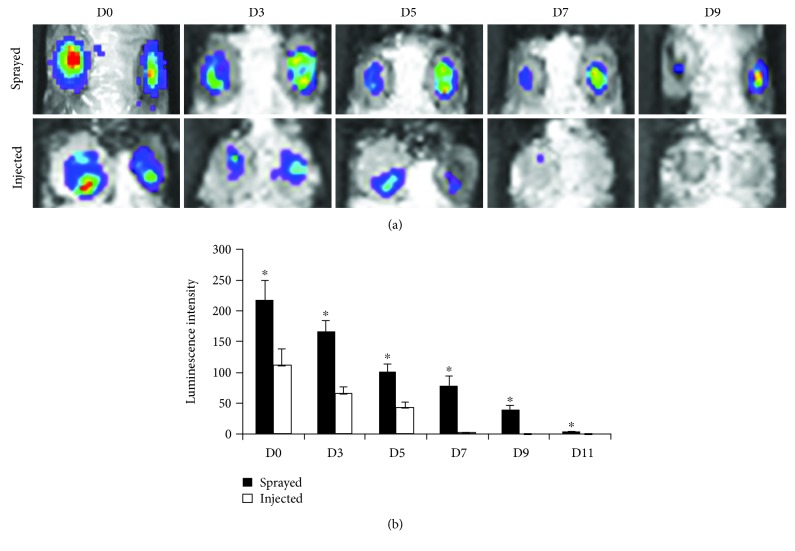
Fibrin significantly prolongs ASC survival when being delivered to diabetic wounds. Cell survival was tracked by an IVIS system and measured fluorescence signal at days 0, 3, 5, 7, 9, and 11 retrospectively. While no fluorescence signal could be found in the control group from day 7 on, fibrin group showed fluorescence emission until day 11.

When comparing luciferase-induced ASCs that were sprayed with fibrin glue onto the murine wound bed, to those that were injected with PBS, we found significantly enhanced ASC levels at days 0, 3, 5, 7, and 9 (*p* < 0.01). In the PBS control group, no fluorescence signal could be detected from day 9 on. Contrarily, ASCs in the fibrin group showed survival past day nine (Figure 2).

### 3.3. Fibrin-Delivered ASCs Significantly Enhance Wound Healing

**Figure 3:**
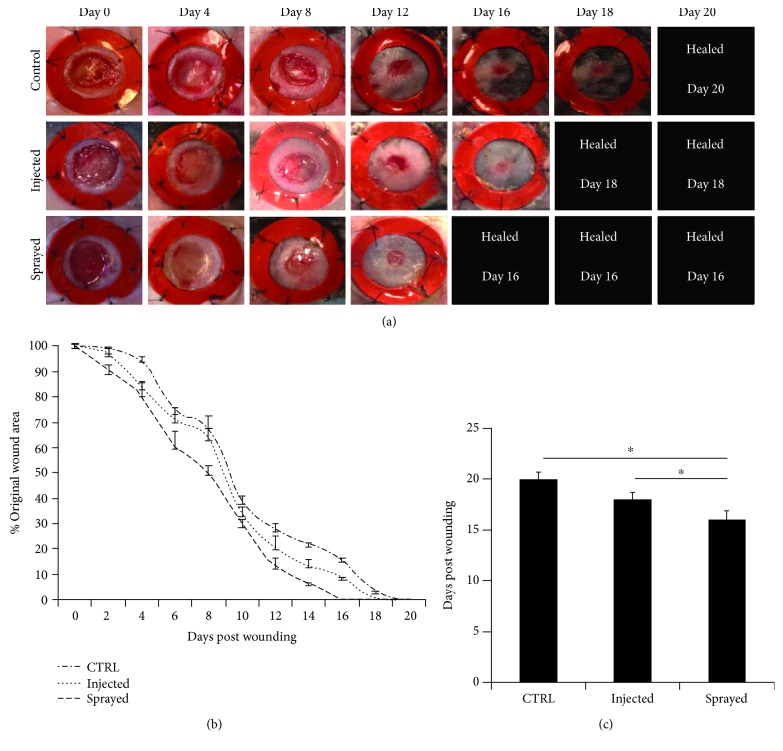
Murine diabetic wounds healed significantly faster in the fibrin group then in the injected and the control group. Wounds were monitored until complete wound closure on days 0, 4, 8, 12, 16, 18, and 20 retrospectively. While sprayed wounds showed complete closure after a mean of 16 days, wounds that were treated with injected ASCs healed after an average of 18 days and control group after 20 days. Complete wound closure was achieved significantly faster in fibrin group compared to the others.

Fibrin glue plus ASCs significantly accelerated wound-healing time in a murine model compared to control groups. Wounds in the fibrin/ASC group healed in a mean of 16 days compared to a mean of 18 days in the ASC injection group (*p* < 0.05) and a mean of 20 days in the fibrin only group (Figure 3) (*p* < 0.05).

### 3.4. Fibrin-Delivered ASCs Enhance Neovascularization Compared to Injected ASCs

**Figure 4:**
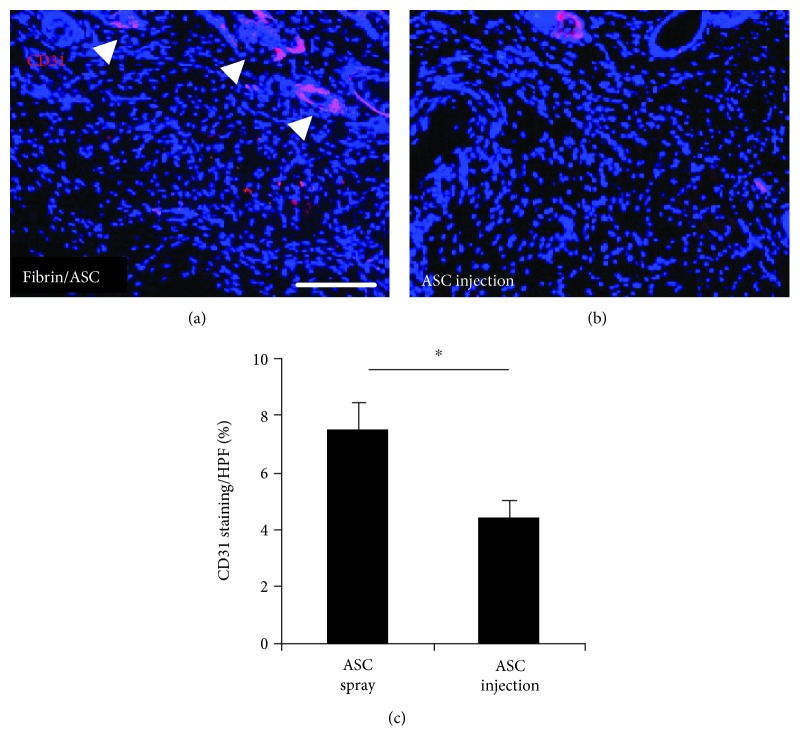
Immunohistochemistry for CD31 after complete wound closure. After complete wound closure, we measured CD31 as a marker for neovascularization and found significantly enhanced levels in the fibrin group (mean, ±SEM) than in the injected group (mean, ±SEM) and in the control group.

When comparing the total fluorescence of immunohistological pictures (high-power field of view = HPF, Figure 4), we found a significant enhancement of CD31 expression in the fibrin/ASC group compared to the ASC injection group. Wounds injected with ASCs in PBS had a total fluorescence of 4.27% while wounds in the fibrin/ASC group showed 7.41% fluorescence (*p* < 0.05).

## 4. Discussion

Although ASCs represent a promising wound-healing therapy, their clinical use is still limited due to variability in efficacy. As described in some of our previous studies, slight differences in harvesting and processing have great influence on differentiation, viability, and cytokine secretion of ASCs [7, 25–27]. Moreover, patient-specific factors contribute to the unpredictability of clinical success. In addition to differences in the wound bed and healing capacity between patients, ASC function and viability are also dictated by age, gender, and comorbidities such as diabetes [3, 9, 12, 13, 28–32]. Although addressing these comorbidities would be the ideal solution, enhancing the niche in which these cells reside is an alternative and elegant strategy.

Despite excellent results both *in vitro* and *in vivo*, to date, the various approaches and vehicles employed for ASC delivery have not been translated to clinical practice [8, 16, 18]. This may, in large part, be due the fact that most of these carrier vehicles have been designed specifically for research proposes and are not freely available in the clinic. To overcome this problem, we utilized a product for delivering ASCs that is already in broad clinical use. Therefore, regulatory issues related to the vehicle can easily be avoided. Although its ability as a cell carrier is rarely described, its clinical safety has been proven extensively and is readily available in most hospitals. Furthermore, fibrin glue is often used to improve skin graft adherence, particularly in the setting of large burns and could enhance outcomes in these patients.

When considered in tandem with existing studies that have shown its clinical benefits [33], our mechanistic study demonstrates a significant enhancement of ASC wound-healing capacity when provided with a fibrin glue niche. The fibrin glue significantly enhanced the secretion of SDF-1 and bFGF, regenerative cytokines that enhance wound healing by improving the recruitment of circulating cells, supporting fibroblast function and promoting neovascularization [34–36].

MMP-2 is a hypoxia responsive cytokine responsible for matrix degradation [37]. Although some reviews describe the stimulation of keratinocyte migration by MMP-2, other murine as well as human trials have shown a significant beneficial effect of selective MMP-2 inhibition on wound closure time [38, 39]. Additionally, high MMP-2 levels in human wound fluid have been proven to be associated with the occurrence of nonhealing wounds [40]. ASCs delivered in fibrin glue have the potential to enhance wound healing by significant upregulation of proregenerative cytokines and simultaneous downregulation of pivotal inhibitors of wound healing. Fibrin glue thereby represents a scaffold-like delivery vehicle that can counteract the harsh wound milieu. Additionally, going along with previous studies, we strongly agree that direct injection of cells in tissue or topical application can significantly decrease cellular function either by mechanical forces or by limited cell adhesion [23].

To drive clinical translation, we confirmed these findings in a well-established murine diabetic wound model [7, 8, 12, 13, 25, 26, 41–43] in combination with an IVIS system and luciferase-marked ASCs, allowing us to track *in vivo* cell viability over time. While the harsh wound environment with diminished blood and nutritional supply rapidly depleted the injected ASCs, the fibrin niche prolonged ASC survival. In addition, to improve the cytokine profile of ASCs, the fibrin glue maintained the benefit of these enhanced ASCs within the wound environment for an extended period of time. Collectively, these effects improved neovascularization and accelerated wound closure.

## 5. Conclusion

Although ASCs represent the most promising approach in regenerative medicine, its clinical use is still limited by uncertainty. The combination of fibrin glue and ASCs represents a reliable, safe, and easy way to enhance stem cell function, viability, and in vivo wound closure time. It also has implications for other clinical settings in which fibrin glue is routinely used, such as large burns.

## Data Availability

The data used to support the findings of this study are included within the article.
